# Multi‐Surface Adhesion Luminescent Solar Concentrators for Supply‐Less IoT

**DOI:** 10.1002/advs.202400540

**Published:** 2024-07-15

**Authors:** Gonçalo Figueiredo, Sandra F. H. Correia, Bruno P. Falcão, Vitor Sencadas, Lianshe Fu, Paulo S. André, Rute A. S. Ferreira

**Affiliations:** ^1^ Department of Physics and CICECO – Aveiro Institute of Materials University of Aveiro Aveiro 3810‐193 Portugal; ^2^ Department of Electrical and Computer Engineering and Instituto de Telecomunicações Instituto Superior Técnico University of Lisbon Lisbon 1049‐001 Portugal; ^3^ Instituto de Telecomunicações and University of Aveiro Campus Universitário de Santiago Aveiro 3810‐193 Portugal; ^4^ Department of Materials and Ceramic Engineering and CICECO – Aveiro Institute of Materials University of Aveiro Aveiro 3810‐193 Portugal

**Keywords:** building‐integrated photovoltaics, Internet of Things, luminescent solar concentrators, self‐adhesive, temperature sensors

## Abstract

The growing prevalence of Internet of Things (IoT) devices hinges on resolving the challenge of powering sensors and transmitters. Addressing this, supply‐less IoT devices are gaining traction by integrating energy harvesters. This study introduces a temperature sensor devoid of external power sources, achieved through a novel luminescent solar concentrator (LSC) device based on a stretchable, adhesive elastomer. Leveraging a lanthanide‐doped styrene‐ethylene‐butylene‐styrene matrix, the LSC yielded 0.09% device efficiency. The resultant temperature sensor exhibits a thermal sensitivity of 2.1%°C^−1^ and a 0.06 °C temperature uncertainty, autonomously transmitting real‐time data to a server for user visualization via smartphones. Additionally, the integration of LED‐based lighting enables functionality in low‐light conditions, ensuring 24 h cycle operation and the possibility of having four distinct thermometric parameters without changing the device configuration, stating remarkable robustness and reliability of the system.

## Graphical abstract

A self‐powered temperature sensor is fabricated through a novel large‐area luminescent solar concentrator (LSC) based on an adhesive lanthanide‐doped elastomer, with thermal sensitivity and temperature uncertainty of 2.11%°C^−1^ and 0.007 °C, respectively, transmitting real‐time data to a server for user visualization. The prototype also includes LED‐based artificial lighting system, ensuring 24 h cycle operation.

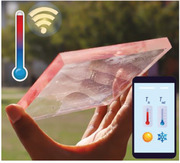

## 1 Introduction

**Figure 1:**
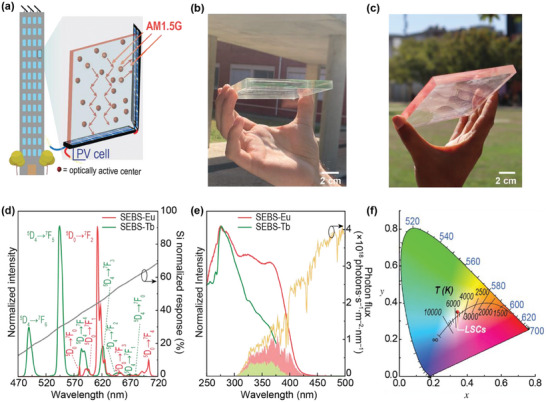
a) Schematic representation of the building integrated LSCs attached to Si PV cells. The red arrows indicate the total internal reflection of the emitted light. Adapted with permission under the terms of the Creative Commons Attribution License (https://creativecommons.org/licenses/by/4.0/).^[^ ^2^ ^]^ Photographs of the LSC based on b) SEBS‐Tb and c) SEBS‐Eu under natural illumination. d) Emission spectra of SEBS‐Eu and SEBS‐Tb excited at 310 and 370 nm, respectively. The grey line (right *y‐axis*) is the normalized Si absorption curve. e) Excitation spectra of SEBS‐Tb and SEBS‐Eu monitored at 544 and 612 nm, respectively. The right *y*‐axis represents the AM1.5G photon flux and the shadowed area is the overlap integral *O*. f) CIE 1931 color space diagram showing color coordinates of the transmitted light through the LSCs under AM1.5G radiation.

By 2025, an estimated 27 billion Internet of Things (IoT) devices will emerge, demanding power sources suitable for compact, hard‐to‐reach installations.^[^ ^1^ ^]^ Energy harvesting, particularly through luminescent solar concentrators (LSCs, **Figure** **1**a),^[^ ^2^ ^]^ stands as a promising solution. LSCs, utilizing photon‐to‐electricity conversion, have shown potential since their conception in the 1970s,^[^ ^3^, ^4^, ^5^ ^]^ yielding optical and power conversion efficiencies up to 18.8% and 7.1%, respectively.^[^ ^6^, ^7^, ^8^, ^9^ ^]^ While current LSC prototypes are mostly small‐scale,^[^ ^10^ ^]^ recent efforts focus on scaling up to sizes over 100 cm^2^.^[^ ^11^, ^12^, ^13^, ^14^, ^15^, ^16^, ^17^, ^18^, ^19^, ^20^, ^21^, ^22^ ^]^ One of the light key parameters, especially when working with large‐area devices with long optical paths, is the reabsorption losses arising from the overlap between absorption and emission.^[^ ^7^ ^]^ Lanthanide (Ln^3+^)‐based complexes stand out due to the ability to engineer a ligands‐induced Stokes‐shift, minimizing this effect.^[^ ^23^ ^]^ Moreover, they offer tunable absorption and emission properties, facilitating photon‐to‐electricity conversion for photovoltaic cells from different technologies and materials. We note that perovskite and CuInS_2_ quantum dots (QDs) serve as highly relevant optically active centers within the LSC field,^[^ ^10^, ^24^, ^25^ ^]^ their synthesis procedures tend to be more intricate compared to those of Ln‐based materials, occasionally leading to safety and toxicity concerns. Furthermore, the emission quantum yield values typically observed in such materials applied to LSCs drift ≈80%,^[^ ^10^, ^26^, ^27^, ^28^, ^29^, ^30^, ^31^, ^32^, ^33^, ^34^, ^35^, ^36^, ^37^ ^]^ a figure attainable with Ln‐based materials.^[^ ^23^, ^38^, ^39^, ^40^ ^]^ In addition, the knowledge of the emission mechanisms in Ln‐based LSC arising from the intra‐4f transitions is an added benefit as the modeling is easily applied, despite recent advances in the use of artificial intelligence (AI) and machine learning approaches that provide evidence to predict LSC performance for distinct optically active centers, including QDs.^[^ ^10^, ^24^, ^25^ ^]^ Advancements now integrate sensing capabilities into LSCs, enabling them to function as sunlight‐powered optical temperature sensors connected to IoT networks.^[^ ^2^ ^]^ This approach, leveraging luminescence for temperature measurement, has evolved from traditional spectrometry^[^ ^41^, ^42^, ^43^ ^]^ to mobile optical sensing using smartphone cameras.^[^ ^44^, ^45^ ^]^

Incorporating luminescent species into polymer matrices, such as styrene‐ethylene‐butylene‐styrene (SEBS), ensures processability and photostability. SEBS is an elastomer with outstanding stability to UV radiation and excellent weatherability which makes it suitable for outdoor applications. Moreover, it is a very flexible and elastic material, with gluey properties allowing its adhesion to virtually any surface.^[^ ^46^ ^]^ The hydrophobic nature of SEBS shields against leaks and enhances luminescence from Ln^3+^‐based complexes dispersed in the polymer matrix. Carefully designed luminescent complexes – sensitized by specific organic ligands – exhibit high quantum yields and UV excitation features, making them compatible with SEBS host matrices for LSC fabrication.

Sunlight intermittence and the need for adequate visible light transmittance (transparency) pose significant challenges for LSCs, crucial for occupant comfort in buildings. Transparent films absorbing UV/blue light while emitting infrared light without affecting natural daylight are ideal. However, NIR LSCs suffer lower efficiencies due to intrinsically limited emission quantum yield.^[^ ^27^, ^29^, ^47^ ^]^ The former aspect can be explored through LEDs coupling to the edges of the LSC to make them work with artificial indoor lighting.^[^ ^48^, ^49^ ^]^

This work introduces supply‐less IoT‐based windows using large‐area LSCs with Ln^3+^ flexible SEBS films, marking the first instance of LSC prototypes using Ln^3+^‐based complexes with surface area above 100 cm^2^ (Figure 1b,c).^[^ ^45^ ^]^ Coupling the LSC with UV and white LEDs enables continuous 24 h operation and definition of four distinct thermometric parameters without changing the system configuration, significantly boosting its performance, robustness, and trustworthiness. Taking advantage of the double‐glazed window concept, the indoor and outdoor temperatures can be simultaneously accessed. Additionally, the thermal sensitivity of the luminescent films enables real‐time mobile temperature sensing^[^ ^45^, ^50^ ^]^ without extra devices or power consumption, which could help optimize heating/cooling systems in buildings.

## 2 Results and Discussion

### 2.1 Structural and Optical Characterization of the Luminescent Materials

Structural characterization based on FT‐IR spectra of SEBS and SEBS doped with different concentrations of Eu‐ or Tb‐based complex (Figure S1a, Supporting Information) presents vibrational bands at 2960 and 2853 cm^‒1^ attributed to the asymmetric and symmetric stretching vibrations of the CH_2_ groups, respectively, of all polymer blocks (styrene, ethylene and butylene), whereas the band at 2921 cm^‒1^ belongs to C─H stretching vibration on saturated carbon.^[^ ^51^ ^]^ The strong vibration bands at 1460 and 1380 cm^‒1^ are associated with the C−H in‐plane bending vibration and the C─H bending in CH_3_ groups, respectively. The vibrational bands at 698 and 762 cm^‒1^ are ascribed to the C−H bending of the monosubstituted benzene ring. To investigate the Ln interaction with the SEBs host, the FT‐IR spectra of Eu‐ or Tb‐based complex are presented in Figure S1a,b (Supporting Information). It was noticed that the SEBS‐Tb and SEBS‐Ln present weak vibrational bands at 1600 and 1275 cm^‒1^, which may be related to the vibrations from Eu‐ and Tb‐based complexes (≤ 1 wt%), respectively.

The XRD pattern of SEBS (Figure S1c,d, Supporting Information) shows a broad band centered at 18.5° with some small diffraction peaks, corresponding to monoclinic crystals (α‐form).^[^ ^52^ ^]^ After the incorporation of lanthanide compounds, there are no significant changes in the diffraction profiles for the Tb‐based complex with different doping contents, but evident changes in the case of the incorporation of the Eu‐based complex, implying that it may affect the structure of SEBS (Figure S1b, Supporting Information).

The emission spectra of the Tb(BBA)_3_Phen complex show the characteristic ^5^D_4_→^7^F_6‐0_ transitions,^[^ ^53^ ^]^ whereas for Eu‐TFNB‐Phen the ^5^D_0_→^7^F_0‐4_ transitions^[^ ^54^ ^]^ are observed (Figures S2 and S3, Supporting Information). After the complexes were incorporated into SEBS, the luminescent polymers emit bright green and red colors, respectively. Figure 1d shows the room temperature emission spectra of the SEBS‐Tb and SEBS‐Eu samples excited at the wavelength that maximizes the emission intensity. Similarly to the isolated complexes, the emission spectra of SEBS‐Tb and SEBS‐Eu are dominated by the ^5^D_4_→^7^F_6‐0_ and ^5^D_0_→^7^F_0‐4_ transitions, respectively. Also, in terms of energy, relative intensity, full‐width‐at‐half‐maximum, and number of Stark components, these spectra resemble those observed for the isolated complexes pointing out that these compounds preserve their local structure after blended with SEBS. Independently of the selected excitation wavelength (275–380 nm), no sign of the SEBS intrinsic emission (Figures S4–S8, Supporting Information) could be observed, suggesting efficient SEBS‐to‐ligand/Ln^3+^ energy transfer.^[^ ^55^ ^]^

The emission is visible to the naked eye due to the high absolute emission *quantum yield* values (*q* = 0.54 ± 0.05 and q = 0.59 ± 0.06 for the SEBS‐Eu and SEBS‐Tb, respectively), which are among the largest ones known for Ln‐doped polymers^[^ ^10^, ^53^, ^56^, ^57^ ^]^ and higher than those of the isolated complexes (Table S1, Supporting Information), indicating compatibility between the SEBS matrix and Ln^3+^‐based complexes. The ^5^D_4_ and ^5^D_0_ states reveal typical lifetime values ≈0.786 ± 0.001 and 0.544 ± 0.001 ms, respectively (Figures S9–S15, Supporting Information). The absorption ability is evidenced in the excitation spectra monitored around the more intense transitions for each case (Figure 1e). Two main components peaking at 330 and 360/390 nm ascribed to the *π–π* ^*^ electronic transition of the Phen ligand,^[^ ^58^ ^]^ overlap the AM1.5G solar irradiation and the emission of UV commercial LEDs. In particular, the overlap integral *O* between the excitation spectra and AM1.5 is ≈0.5% and ≈1.5% of the solar photon flux on Earth (4.3 × 10^21^ photons s^−1^ m^−2^) for SEBS‐Tb and SEBS‐Eu, respectively.^[^ ^59^ ^]^ The photostability was tested by exposing the SEBS‐Tb and SEBS‐Eu to accelerated aging tests performed in a climatic chamber showing that these samples are stable under extreme conditions, with a relative deviation on the *q* values below 1%.

### 2.2 Performance of Fabricated Luminescent Solar Concentrators

To demonstrate the processability and applicability of the SEBS‐Tb and SEBS‐Eu materials, planar LSCs were fabricated by coating 10.5 × 10.5 × 0.8 cm^3^ glass substrates (Figure 1b,c, respectively). In both cases, it is possible to observe a bright green or red color (Figure S17a, Supporting Information). As transparency of LSC devices is a prime aspect for indoor applications since it directly impacts on aesthetics and visual comfort,^[^ ^60^ ^]^ the quality of the transmitted light was assessed by the average visible transmission (AVT) parameter, which is dependent on the photopic response of the human eye:^[^ ^61^, ^62^, ^63^ ^]^

(1) $$AVT=\frac{\int T\left(\lambda \right)P\left(\lambda \right)\mathrm{AM}1.5G\left(\lambda \right)d\left(\lambda \right)}{\int P\left(\lambda \right)\mathrm{AM}1.5G\left(\lambda \right)d\left(\lambda \right)}$$

where 𝑇(𝜆) is the transmission spectra (Figure S18, Supporting Information), *P*(𝜆) is photopic response of the human eye and AM1.5G(𝜆) is the solar photon flux spectrum (photons s^−1^ m^−2^). This parameter is a measure of how much light people will perceive as passing through the LSC. The AVT of the fabricated planar LSCs was found to be ≈90%, considered acceptable for window‐related applications.^[^ ^61^, ^62^ ^]^ To quantify the color appearance of the planar LSCs and their effect on color perception, the transmitted light was analyzed in terms of Commission Internationale d’Éclairage (CIE, 1931) color space diagram coordinates and color rendering index (CRI) which evaluated the ability to accurately render the color of objects. As presented in Figure 1f, the light transmitted through the LSCs shows coordinates (0.34, 0.35) very close to ideal white light (0.33, 0.33). The CRI is evaluated on a 0–100 scale, with a CRI above 70 being considered good quality and above 95 of excellent quality.^[^ ^61^, ^64^ ^]^ In the case of the fabricated LSCs, CRI values of 97 for both the planar LSCs based on SEBS‐Tb and SEBS‐Eu, respectively, reveal nondistortion of incoming sunlight. Also confirming these features are the found color correlation temperature values (CCT) of the transmitted light (5127 K) which are very close to the incoming light (5161 K), as shown in Figure 1f.

**Figure 2:**
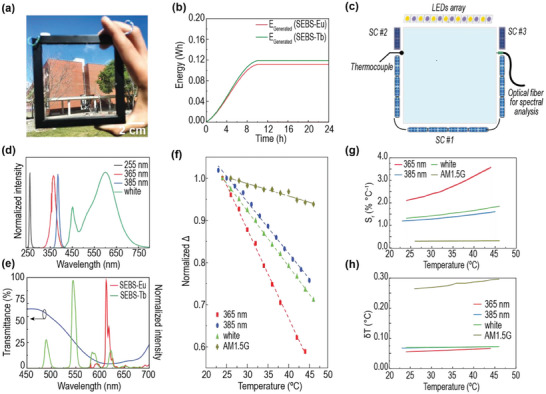
a) Photograph of the LSC based on SEBS‐Eu under natural illumination assembled with PV cells showing its transparency. b) Energy generated by the LSC devices. c) Scheme of the LSC prototype for temperature‐dependent measurements, showing the artificial lighting system and the several solar cells used to estimate the thermometric parameter. d) Emission spectra of the LEDs. e) Transmittance spectra of the high pass filter with SEBS‐Tb and SEBS‐Eu emission spectra. f) Normalized thermometric parameter Δ for the SEBS‐Eu prototype according to the illumination source (the dashed lines are the best linear fit with r^2^>0.99). g) Relative thermal sensitivity *S_r_* calculated using Equation 1; h) temperature uncertainty *𝛿T* calculated using Equation 2.

The LSCs performance was evaluated by coupling an array of Si PV cells (**Figure** **2**a; Figure S16, Supporting Information) and characterized under simulated AM1.5G irradiation, yielding external photon conversion efficiency (*η*_ext_) and device efficiency (*η*_dev_) values (Supporting Information for details) of 3.3% and 0.08%, respectively, for the LSC based on SEBS‐Tb and 2.6% and 0.09%, for the LSC based on SEBS‐Eu. The similar performance of the LSCs was further explored by estimating the devices’ output energy considering an initial 24 h period with a maximum recorded solar radiation of 1047 W m^−2^ after four hours from the start of the test, showing analogous results (Figure 2b). When evaluating LSC performance, it should be noted that the *η*_dev_ values depend on the type of PV cell technology employed in the LSC‐PV system as the power generated by the LSC device is heavily influenced by the electrical characteristics of the PV device.^[^ ^65^, ^66^ ^]^ Therefore, focusing on the *η*_ext_ values, which is the figure of merit that accurately reflects the performance of the LSC as a photonic device,^[^ ^65^ ^]^ our devices are among the highest values reported for large‐area planar LSCs (Table S2, Supporting Information).^[^ ^10^ ^]^

### 2.3 Temperature Sensors Based on LSCs

The temperature‐dependence of the photoluminescence features of Tb‐ and Eu‐doped materials was already reported and used to fabricate luminescent thermometers, and thus it is expected that the optical and electrical performances of the LSCs will also vary as a function of the temperature under solar simulator irradiation.^[^ ^2^, ^67^ ^]^ The electrical performance of the PV cells coupled to the LSCs was analyzed as a function of the temperature and a decrease of the generated electrical power (P_out_) with increasing temperature was verified.

For the LSC sensor prototype testing, the setup presented in Figure 2c was used, including an artificial lighting system composed of LEDs and several PV cells, to feed the system and to monitor temperature in solar intermittence conditions. The artificial lighting system is composed of UV‐emitting LEDs for selective excitation of the luminescent materials (255 and 365 nm for LSCs based on SEBS‐Tb and SEBS‐Eu, respectively) and white‐emitting LEDs to simulate indoor illumination (Figure 2d). The key voltage to estimate the thermometric parameter and feed the IoT system is provided by the main solar cells array (SC #1). As shown in Figure 2c, two reference solar cells (SC #2 and SC #3) covered by a high pass filter able to discriminate the photons from the luminescent material emission to those from the original light source (Figure 2e) were also included (details in the Experimental Section and Supporting Information). This approach allowed the implementation of a ratiometric thermometer, which is less prone to external influences.

For the illustrative case of the SEBS‐Eu prototype, as a proof‐of‐concept, the electrical performance of the coupled PV cells was analyzed as a function of the temperature, and a thermometric parameter based on the open‐circuit voltage (*V*_oc_) was implemented:

(2) $$\Delta \;=\frac{V_{oc3}}{V_{oc1}}$$

where *V*_oc1_ and *V*_oc3_ are the open‐circuit voltage values at SC #1 and SC #3, respectively. For indoor applications, Δ was evaluated using the LEDs. For all the illumination sources, the thermometric parameter follows a linear dependence with temperature (Figure 2f) with S_r_ values up to 2.1 ± 0.03% °C^−1^, and with δT up to 0.06 °C at 25 °C (Figure 2g,h; Table S3, Supporting Information) which grants the possibility to accurately sense temperature. It is worth to highlight that the small values of δT here reported are due to the resolution of the equipment used to measure *V*_oc1_ and *V*_oc3_ values. The existence of 4 thermometric parameters in the same window without the need for modification ensures the robustness of the measurement and contributes to its reliability, ensuring multi‐readout.

A practical example of applicability was performed through the development of an IoT system implemented using a programmable board to measure the *V*_oc1_ and *V*_oc3_ values (Supporting Information for details). The voltage values and the estimated temperature are sent by Wi‐Fi to an IoT analytics platform service allowing aggregation, visualization, and analysis of real‐time data streams. The overall sensor electrical output is enough to use part of the PV cell's generated electrical current for the measurements and the remaining current is used to power the IoT system, constituting a supply‐less system arising from the fact that the whole sensing and IoT device (Figures S19 and S20, Supporting Information) is powered by the LSCs.

### 2.4 Large‐Scale Prototype

**Figure 3:**
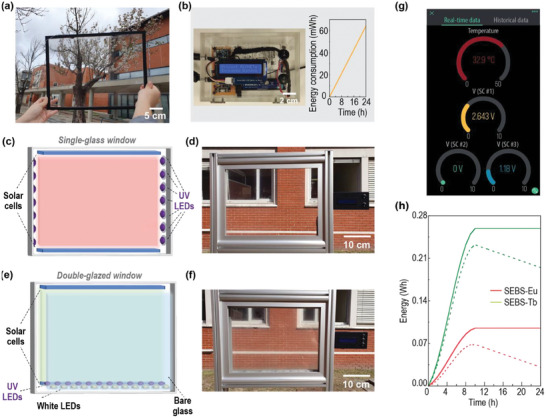
Photographs of the a) large‐scale prototype based on SEBS‐Eu under natural outdoor illumination and of the b) IoT electronics box (the inset shows the energy consumed by the components of the IoT system used to measure and transmit the data from the LSC prototypes). c) Scheme and d) photograph of the SEBS‐Eu LSC prototype assembly as a single‐glass window. e) Scheme and f) photograph of the SEBS‐Tb LSC prototype as a double‐glazed window for SEBS‐Tb LSC. d,f) The photographs show the prototypes in the final demonstrator, including the LSC devices and the IoT system. g) Reproduction of a screenshot of the *Blynk* platform showing voltage and temperature values. h) Energy generated by the LSCs (solid lines) and net energy to be stored in energy production units (dashed lines).

A large‐scale prototype was fabricated, to simulate a solar window with temperature‐sensing ability. Thus, LSC prototypes with dimensions of 28 × 38 × 0.8 cm^3^ (**Figure** **3**a) were fabricated and connected to the IoT (Figure 3b). Two configurations were included in the construction of the final demonstrator: i) a single‐glass window based on SEBS‐Eu (Figure 3c,d), where the solar cell arrays and the LEDs for artificial lighting are coupled to the edges, and ii) a double‐glazed window where the inner glass is coated with SEBS‐Tb (facing inward) and the outer glass remains uncoated, to create a thermal barrier and physical protection of the luminescent layer (Figure 3e,f). This latter configuration allows an enhanced thermal efficiency of the window and the measurement of the temperature inside and outside the building. The PV cell arrays are coupled to the longer edges of the LSCs, and the artificial lighting is placed on the gap between the two glasses. The final demonstrator of the smart energy‐generating window is composed of a polished aluminum structure (Figure S21, Supporting Information) assembled to frame both large‐scale prototypes and all the electronic components associated with the IoT platform, including an interface where the user may visualize the temperature data (Figure 3g). The energy balance was also measured for the large‐scale prototypes, in a scenario where artificial lighting was switched off and considering the 24 h energy consumption shown in the inset on Figure 3b. The value of the energy consumed by the circuit was measured in five‐minute cycles and is the result of the energy needed to keep the circuit active for a period of twelve seconds for data acquisition and transmission and the energy required to keep the circuit in minimum energy consumption mode for the rest of the cycle. The cumulative energy generated by the SEBS‐Eu prototype and the SEBS‐Tb prototype are not directly compared (as in Figure 2) due to the distinct geometry. In both cases, this energy rises during daylight hours and remains steady during the evening hours (Figure 3h). Based on the net energy of the prototypes over the initial 24 h period (Figure 3h), there is no additional need for an external power supply.

## 3 Conclusion

A large‐scale LSC prototype based on a lanthanide‐doped polymeric matrix with temperature‐sensing ability is reported. The external photon efficiency and the device efficiency values for the representative case of a europium‐based device were found to be 2.6% and 0.09%, respectively. The temperature sensor was established based on the electrical output of the coupled photovoltaic cells, yielding maximum values of thermal sensitivity and temperature uncertainty of 2.1%°C^−1^ and 0.06 °C, respectively, under LED illumination. Full demonstrators able to work with natural sunlight illumination and artificial lighting (UV‐ or white‐emitting LEDs) composed with a single‐glass window or a double‐glazed window were assembled and tested, proving the suitability for a real‐life application, holding promise for optimizing building heating/cooling systems, thereby enhancing energy efficiency and savings, and ultimately, improving thermal efficiency.

## 4 Experimental Section

### Materials

The selected luminescent complexes were designed by considering the quantum yield, excitation wavelength and compatibility with the host SEBS. As the organic ligands 4,4,4‐trifluoro‐1‐(2‐naphthyl)‐1,3‐butanedione (TFNB) and 4‐benzoyl benzoic acid (BBA) can sensitize the luminescence for Eu^3+^and Tb^3+^, respectively, through the strong absorption from the ligands and efficient energy transfer from the ligands to Ln^3+^ ions, and the auxiliary ligand 1,10‐phenanthroline (Phen) can further improve the luminescence feature, the Ln^3+^‐complexes used in this work show high quantum yields (0.35±0.04 for Eu^3+^‐based complex and 0.28 ± 0.03 for Tb^3+^‐complex), near‐UV excitation features, and can be combined well with SEBS for LSC fabrication.

### Structural Characterization

Powder X‐ray diffraction (XRD) patterns were recorded in the 2θ range of 3.0–60.0° on Panalytical Empyrean Diffractometer under exposure to CuKα radiation (λ = 1.5418 Å) at room temperature. The Fourier transform infrared (FT‐IR) spectra for the ligands and the complexes were acquired technique with 64 scans and 2 cm^−1^ resolution on JASCO FT/IR‐4X FTIR Spectrometer using KBr pellet. The FT‐IR‐ATR spectra for the SEBS and the doped luminescent materials were obtained by 256 scans and 4 cm^−1^ resolution on Tensor 27 spectrometer (Bruker) with an ATR‐IR accessory (“Golden Gate”, Specac, Ltd.).

### Optical Characterization

The photoluminescence spectra were recorded with a modular double‐grating excitation spectrofluorimeter with a TRIAX 320 emission monochromator (Fluorolog‐3, Horiba Scientific) coupled to a R928 Hamamatsu photomultiplier. The *q* values were measured at room temperature using a system (C9920‐02, Hamamatsu) with a 150 W xenon lamp coupled to a monochromator for wavelength discrimination, an integrating sphere as the sample chamber, and a multichannel analyzer for signal detection. The method is accurate to within 10%. Optical parameters of the transmitted light were recorded using an integrating sphere (ISP 150L, Instrument Systems) connected to a detector (MAS40‐121, Instrument Systems), with an integration time of 10 s. Photostability of the SEBS‐Eu and SEBS‐Tb samples was evaluated after being placed inside a climatic chamber (Angelantoni Industrie, model Challenge 340) subjected to a temperature of 50.0 °C and 95% relative humidity. The accuracy is 0.3 °C and 3%, respectively. The test had the duration of 24 h. The integrating sphere above mentioned was used for the measurement of the emission quantum yield.

### Luminescent Solar Concentrators

The Eu‐ and Tb‐doped SEBS samples (synthesis details can be found in Supporting Information) were deposited on glass substrates (10.5 × 10.5 × 0.8 cm^3^ or 28 × 38 × 0.8 cm^3^) using the doctor‐blade deposition method (Automatic Film Applicator AB4400, TQC Sheen). LSCs prototypes with dual‐photoluminescence layers were also fabricated by coating each side of the glass substrate with the Eu and Tb‐based solutions (Figure S19b, Supporting Information). The 10 × 10 × 0.8 cm^3^ LSC prototypes were framed in polyvinyl chloride (PVC), in which an array of 12 solar cells of c‐Si connected in series (IXOLAR^TM^ SolarBITs, ANYSOLAR, KXOB25‐01×8F) was attached to collect light in three edges of the prototype (Figure S16a, Supporting Information). The UV LEDs emitting at 255 and 365 nm were coupled to the edge of the LSCs based on SEBS‐Tb and SEBS‐Eu, respectively. The *I–V* characteristic of all prototypes was measured at room temperature under simulated AM1.5G radiation (OSRAM Ultra‐Vitalux 300 W, ≈225 W m^−2^). Additionally, the *I–V* data were recorded when exposed to the illumination of an array of 5 white LEDs (Lumileds LUXEON 3030 HE Plus, CCT 4000 K, CRI 80) placed on one edge of the prototype (Figure S16b, Supporting Information). The energy output measurements were performed outdoors in which the solar irradiance values were monitored in real‐time (with a 10 min interval) using data obtained from the weather station located in Portugal (Aveiro)^[^ ^23^ ^]^ or a calibrated reference solar cell (91150 V, Newport).

### Temperature‐Dependent Measurements

The main solar cells array SC #1 of the LSC sensor prototype was composed of 10 c‐Si solar cells (IXOLAR, KXOB25‐14×1F) connected in series. The SC #2 and SC #3 reference solar cells (IXOLAR KXOB25‐14×1F and KXOB25‐01×8F, respectively) maximized current and voltage values, respectively. For the large‐scale prototype, the solar cell arrays were coupled only on the longer edges of the LSC where SC #1 (IXOLAR, SM281K07TF) and SC #3 (IXOLAR, KXOB25‐01×8F) solar cells were placed. It was noted that a direct performance comparison between small and large‐scale prototypes cannot be performed as due to their distinct geometry and the difference in the PV cells. Temperature‐dependent *I–V* measurements under AM 1.5G illumination were performed in the temperature range of 25–45 °C. Simultaneously, the emission spectra were recorded at the edge of the LSC using an optical fiber connected to a portable spectrometer (SensLine, AVANTES, slit 100 µm) for real‐time acquisition. All measurements were carried out in a homemade setup comprising a temperature‐controlled heat plate, a thermocouple sensor to monitor the local temperature of the prototypes, and a Keithley 2400 series source meter to record the *I–V* values. In this case, to have a calibration curve independent of the power fluctuations of the light source, the Δ values were normalized to the ones at 25 °C. The measurements were performed under distinct irradiation conditions: i) UV‐emitting LEDs at 365 nm, ii) UV‐emitting LEDs at 385 nm, iii) white‐emitting LEDs, and iv) AM1.5G (OSRAM Ultra‐Vitalux 300 W) (Figures S22 and S23, Supporting Information).

## Conflict of Interest

The authors declare no conflict of interest.

## Supporting information

Supporting Information

Supplemental Video 1

Supplemental Video 2

## Data Availability

The data that support the findings of this study are available from the corresponding author upon reasonable request.
