# Supplementary material for: Multi‐Surface Adhesion Luminescent Solar Concentrators for Supply‐Less IoT
Source: Adv Sci (Weinh). 2024 Jul 15;11(35):2400540. doi: 10.1002/advs.202400540 (PMC11425244; doi:10.1002/advs.202400540)
Supplement: Supplementary file 1 — Supporting Information [file ADVS-11-2400540-s002.docx]

Supporting Information

Multi-surface adhesion luminescent solar concentrators for supply-less IoT

*Gonçalo Figueiredo, Sandra F. H. Correia*, Bruno P. Falcão, Vitor Sencadas, Lianshe Fu, Paulo S. André, and Rute A. S. Ferreira**

*sandracorreia@ua.pt; rferreira@ua.pt

1. **Synthesis of optically active layers**

***Materials.*** TFNB (Aldrich) and BBA (Aldrich), Phen (Alfa-Aesar), sodium hydroxide (NaOH), hydrochloride acid (HCl), and ethanol (EtOH) were all commercially available. Europium chloride (EuCl_3_, 0.2 M) and terbium chloride (TbCl_3_, 0.2 M) aqueous solutions were obtained by dissolving europium oxide (Eu_2_O_3_) and terbium oxide (Tb_4_O_7_, Yuelong New Material Co., Ltd., Shanghai, China) in HCl (37%, Aldrich), respectively. The remaining acid was removed by successive evaporation and the resulting solids were dissolved in distilled water. SEBS material (Calprene H6180X, with 85/15 ethylene butylene/styrene ratio) was supplied by Dynasol Group.

***Synthesis of Eu-based complex.*** Eu-based complex was synthesized according to our previous method with some modifications.^[1]^ A mixture of 3 mmol of TFNB and 1 mmol of Phen was dissolved in 20 mL of ethanol (EtOH), then 3 mmol of NaOH aqueous solution was added under stirring. After that, 1 mmol of EuCl_3_ aqueous solution was added. The solution was stirred at 50 °C and the resulting precipitate was filtered, washed with EtOH and n-hexane, dried at 50 °C to get Eu-based complex, and designed as Eu-TFNB-Phen.

***Synthesis of Tb-based complex.*** 1.5 mmol of BBA and 0.5 mmol of Phen were dissolved in 10 mL of EtOH, then 1.5 mmol of NaOH aqueous solution was added under stirring. After that, 0.5 mmol of TbCl_3_ aqueous solution was added under stirring. The resulting precipitate was filtered, washed with EtOH and n-hexane, dried at 50 °C to get to get Tb-based complex, and designed as Tb-BBA-Phen.

***Synthesis of Eu- and Tb-doped SEBS****.* The thermoplastic elastomer SEBS was dissolved in chloroform using a ratio polymer/solvent of 1/9 (w/v), and placed in a magnetic stirrer (MST, VELP) until a clear solution was obtained. The composite samples with different amounts of Eu-TFNB-Phen and Tb-BBA-Phen complexes where prepared by firstly placing the desired amount of the complex in the solvent, followed by dispersion in a ultrasound bath (Sonorex from Bandelin) during 4 h. Afterwards, the polymer was added to the solution and placed in a magnetic stirrer until a complete polymer dissolution. Finally, the solution was cast with the help of automatic film applicator (AB4400, TQC Sheen) and dried at 40ºC in a drying chamber (VDL 56, Binder). The resulting Eu- and Tb-doped SEBS materials will be hereafter designated as SEBS-Eu and SEBS-Tb, respectively. The resulting Ln (Eu, Tb) complexes doped SEBS materials will be hereafter designated as SEBS-Ln-1, SEBS-Ln-2, and SEBS-Ln-3 for 0.25%, 0.5%, and 1.0% (weight ratio) doping contents, respectively. The maximum doping content was set at 1.0% (weight ratio) as further advantages by increasing the doping content are not expected as the emission quantum yield values clearly reached plateau values with this concentration, and the absorption ability can be controlled with the thickness of the film. As the selected concentration to fabricate the LSC prototypes was 1.0%, the designation SEBS-Ln will be used to refer to it.

1. **Prototypes characterization**

The external photon efficiency (*η_ext_*), also commonly known as the optical conversion efficiency (*η_opt_*), was estimated through:^[2]^

$\eta_{ext}\text{=}\frac{\text{P}_{\text{out}}}{\text{P}_{\text{in}}}\text{=}\frac{\text{I}_{\text{SC}}^{\text{L}}\text{V}_{\text{oc}}^{\text{L}}}{\text{I}_{\text{SC}}\text{V}_{\text{oc}}}\frac{\text{A}_{\text{e}}}{\text{A}_{\text{s}}}\frac{\text{η}_{\text{solar}}}{\text{η}_{\text{PV}}}$ (S1)

where $I_{\mathrm{SC}}^{L}$ and $V_{\mathrm{oc}}^{L}$ represent the short-circuit current and the open-circuit voltage when the PV device is coupled to the LSC, $I_{sc}$ and $V_{oc}$ are the corresponding values of the PV device exposed directly to solar radiation, $A_{s}$ and $A_{e}$ are the exposed and total edge areas respectively, $\eta_{solar}$ is the efficiency of the PV device relative to the total solar spectrum and $\eta_{PV}$ is the efficiency of the PV device at the LSC emission wavelengths. Three measurements were performed for each case, with a relative error (${\eta_{ext}}/{\Delta\eta_{ext}}$) found to be below 10 %.

The experimental *η_ext_* values were determined by illuminating the top surface of the LSCs with simulated AM1.5G illumination. The $I_{sc}$ and $V_{oc}$ values were measured using a current source meter device (2400 SourceMeter SMU Instruments, Keithley). The device efficiency (*η_dev_*), which is often called power conversion efficiency (PCE), was calculated through:

${}_{dev}=\frac{P_{out}^{el}}{P_{in}}=\frac{\text{I}_{\text{SC}}^{\text{L}}\text{V}_{\text{oc}}^{\text{L}}FF}{A_{S}\int_{\lambda_{1}}^{\lambda_{2}} I_{AM1.5G}\left( \lambda\right)d\lambda}$ (S2)

where $P_{out}^{el}$ and FF = 0.75 are the PV device output electrical power and fill factor of the PV cell, respectively. The mismatch in the UV spectral region between the AM1.5G solar irradiance and that of the used lamp was considered following a methodology reported in detail elsewhere.^[3]^

The figures of merit to evaluate the performance of the thermometers are the *S_r_* and the temperature uncertainty, *δT*, defined as:^[4,5]^

$S_{r}=\frac{1}{\Delta}\left| \frac{\partial\Delta}{\partial T} \right|$ (S3)

and

$\delta T=\frac{1}{S_{r}}\frac{\delta\Delta}{\Delta}$ (S4)

where δΔ is the uncertainty in the determination of the thermometric parameter Δ.

1. **IoT platform**

The IoT system (Figure S19 in Supporting Information) was implemented in a programmable board (ESP32­WROOM­32D) to measure the *V_oc1_* and *V_oc3_* values, estimate the temperature using the calibration curve, and send the temperature values in real-time to a data server. A voltage sensor module (B25) was included, allowing to measure DC voltage in the 0-25 V range from the SC #1 solar cells array. Also, a battery charger module (TP4056) was included to charge a lithium-ion battery (type 18650, 3.7 V, 2200 mA) and to collect the energy generated by the SC #1 solar cells array. The battery was connected to the charger module using B+ e B- pins, whereas the OUT+ e OUT- pins are the battery output, providing the system with a voltage of $\sim$3.7 V. A voltage regulator was integrated to feed the ESP32­WROOM­32D with 3.3 V from the battery connected to the battery charger module, constituted by a drop regulator, a ceramic capacitor of 100 nF and an electrolytic capacitor of 100 µF. An LSC Grove screen of 16 characters/2lines was also included to allow the user to visualize the estimated parameters in real time.

Figure S19 shows all the IoT electronic components assembled in a box, in which the only connections with the outside are the LED connectors to a controller and the solar cells arrays wiring and the LED controller power cable. Two switches were installed on the external side of the box to turn on/off the LED devices independently. The voltage values and the estimated temperature values are then sent to the programmable bard through wi-fi to the *Blynk* IoT analytics platform allowing an intuitive visualization using a mobile device (Figure 2l in the manuscript).

In the final demonstrator (large-scale prototypes) two individual electronic circuits were implemented on the black boxes placed on the side of each window. Both allow voltage values reading, temperature estimation and data transmission to two distinct platforms: *Arduino Cloud* (for the SEBS-Eu device) and *Ubidots* (for the SEBS-Tb device). The electronic circuits are identical to the ones described above, except that the ESP32-WROOM-32 was replaced by Arduino Nano 33 IoT. Those boards are very popular due to their compact format and IoT functionalities.

1. **Tables**

**Table S1.** Absolute quantum yield (*q*) values of the Ln-based complexes and of the SEBS-Ln materials for excitation wavelength λ_exc_.

| Materials | λ_exc_ (nm) | *q* |
| --- | --- | --- |
| Eu-TFNB-Phen | 395 | 0.35±0.03 |
| Tb-BBA-Phen | 375 | 0.28±0.03 |
| SEBS-Tb-1 | 310 | 0.30±0.03 |
| SEBS-Tb-2 | 305 | 0.49±0.05 |
| SEBS-Tb-3 | 310 | 0.54±0.05 |
| SEBS-Eu-1 | 370 | 0.62±0.06 |
| SEBS-Eu-2 | 370 | 0.61±0.06 |
| SEBS-Eu-3 | 370 | 0.59±0.06 |

**Table S2.** Overview of reported large-area planar LSCs.

| Fluorophore/Host matrix | Dimensions (cm) | *G* | *η_ext_^*^* (%) | *η_dev_^*^* (%) | Ref. |
| --- | --- | --- | --- | --- | --- |
| SEBS-Tb | 10.5×10.5×0.8 | 3.3 | 8.5 | 0.08 | This work |
| SEBS-Eu |  |  | 7.6 | 0.09 |  |
| bPDI-3/LR305/PMMA | 20×20×0.1 | 50 | 1.72 | 0.92 | ^[6]^ |
| 4CzIPN/PMMA | 10×10×0.3 | 8.3 | 5.2  12.6^a)^ | - | ^[7]^ |
|  | 15×15×0.3 | 12.5 | 4.4  9.4^a)^ | - |  |
|  | 20×20×0.3 | 16.6 | 3.7  7.6^a)^ | - |  |
| CISeS/ZnS/PLMA | 12×12×0.3 | 10 | 3.27 | - | ^[8]^ |
| CdSe/CdS/PMMA | 21.5×1.35×0.5 | 1.27 | 1 | - | ^[9]^ |
| CdSe@ZnS/ZnS/pLMA-co-EGDM | 10×9×0.3 | 7.9 | 1.22 | - | ^[10]^ |
| Si QDs/pLMA-co-EGDM | 12×12×0.26 | 11.5 | 2.85 | - | ^[11]^ |
| CdSe/Cd_1-_*_x_*Zn*_x_*S/PVP | 91.4×30.5×nd |  | 3 |  | ^[12]^ |
| Mn^2+^-doped QDs/PVP | 20.32×20.32×0.16 | 31.8 | 6.4 | 3.10 | ^[13]^ |
| PFPBNT/PMMA | 15×15×0.6 | 6.25 | 2.95 | 0.38 | ^[14]^ |
| DTB/DPA/PMMA | 50×50×0.6 | 20.8 | 6.4 | 1.26 | ^[15]^ |
| TPA/BT/PDMS | 10×10×0.2 | 12.5 | 4.2 | 1.4 | ^[16]^ |
| COi8DFI/PBMA | 10.16×10.16×0.3 |  |  | 0.54 | ^[17]^ |
| CuInS_2_/PMMA | 30×30×0.7 |  | 6.8 |  | ^[18]^ |
| CdSe/CdS/PS | 10×10×0.4 | 6.25 | 2.95 | 2.25 | ^[19]^ |
| CDs/PVP | 10×10×1 | 2.5 | 0.92 |  | ^[20]^ |
| CDs/CdSe/CdS-PVP/PLMA | 10×10×0.4 |  | 1.4 |  | ^[21]^ |
| CDs/Perovskite-PVP/PLMA-co-EGDA | 10×10×0.2 | 12 | 3.05 |  | ^[22]^ |
| Perovskite/PS | 10×10×0.4 | 6.25 | 2.4 | 1.8 | ^[23]^ |
| Dye 1-PMMA | 20.32×20.32×0.64 | 5 | 23.7^b)^ | 1.59 | ^[24]^ |
| CdSe/ZnS-PU | 11.5×11.5×0.3 |  |  | 2.1^b)^ | ^[25]^ |
| CDs-PVP | 10×10×0.9 | 1.8 | 1.6 | 0.7 | ^[26]^ |
| CDs-PVP | 15×15×0.5 |  | 2.2 | 1.13 | ^[27]^ |
| Ba^2+^ CDs-PVP | 10×10×0.4 | 2.9 | 3.2 | 1.90 | ^[28]^ |
| yCDs-PVP | 10×10×1 | 2.5 | 4.56 | 4.10 | ^[29]^ |
| Si CDs-PVP | 15×15×0.5 |  |  | 2.06 | ^[30]^ |
| rCDs-PVP | 10×10×0.7 | 3.4 |  | 1.90 | ^[31]^ |
| rCDs-PVP | 10×10×0.9 | 2.5 | 3.0 | 2.7 | ^[32]^ |
| CGAS/ZnS-PLMA-co-EGDA | 20×20×0.15 |  |  | 0.55 | ^[33]^ |
| Lumogen red F-Kraton | 45×50×0.6 |  |  | 0.60 | ^[34]^ |
| rCDs-PMMA | 10×10×0.52 |  | 4.81 | 2.41 | ^[35]^ |
| Lumogen Red 305-PMMA | 60×60×0.3 |  |  | 1.55 | ^[36]^ |
| Perspex acrylic plates | 100×100×0.5 |  |  | 0.20 | ^[37]^ |
| DACT-II-PBzMA | 10×10×0.3 |  | 2.32 | 0.33 | ^[38]^ |
| TPP1-PMMA | 10×10×0.3 | 8.33 | 2.33 | 0.34 | ^[39]^ |
| GQDs-polystyrene | 10×10×0.63 |  | 2.5 | 1.4 | ^[40]^ |
| Si QDs-OSTE | 20×20×nd |  |  | 1.57^b)^ | ^[41]^ |
| Si QDs-octadecene | 12×12×0.4 |  |  | 2.47^c)^ | ^[42]^ |
| CdSe/CdS-PVP | 20×20×0.47 |  | 1.93 |  | ^[43]^ |
| InP/ZnS/Rhod 101-PLMA-co-EGDA | 10×10×0.3 | 8.33 | 1.0 |  | ^[44]^ |
| Cu-deficient CGAS/ZnS-PLMA-co-EGDA | 10×10×0.15 |  |  | 4.29 | ^[33]^ |
| AgInS_2_/ZnS-PLMA | 10.4×10.4×0.2 | 12.98 | 3.8 |  | ^[45]^ |
| CIS/ZnS-PVP | 10×10×1 |  | 1.01 |  | ^[46]^ |
| CuInS2/ZnS-nd | 15.24×15.24×nd |  | 4.36 | 1.87 | ^[47]^ |
| perovskite-PMMA | 10×10×0.2 | 12.5 | 0.87 |  | ^[48]^ |
| perovskite-PMMA | 10×10×0.3 | 12.5 | 2.0 |  | ^[49]^ |
| TPP treated CsPbI_3_- polyester polyurethane acrylate oligomer | 15×5×0.2 | 10 | 3.1 |  | ^[50]^ |
| Cs_2_Ag_0.4_Na_0.6_InCl_6_-PMMA | 10×10×0.2 | 12.5 | 0.7 | 1.5 | ^[51]^ |

* *η_ext_* and *η_dev_* are also known as optical conversion efficiency, *η_opt_*, and powerconversion effcieincy, PCE, respectively; nd=not defined; PMMA= poly(methylmethacrylate); Dye 1= perylene-based dye; 4CzIPN=1,2,3,5 tetrakis(carbazol-9-yl)-4,6-dicyanobenzene; PLMA=poly(laurylmethacrylate); pLMA-co-EGDM= ethylene glycol dimethacrylate copolymer; PVP=polyvinylpyrrolidone ^a)^ measurement performed under white LED illumination using a backside diffuser. ^b)^ using backside reflector. ^c)^ using reflecting mirrors.

**Table S3.** Calibration curve slope (°C^−1^), thermal sensitivity (%°C^−1^) and temperature uncertainty (°C) at 25 °C of the ∆ thermometric parameter for SEBS-Eu LSC prototype.

| Light source | Slope (ºC^-1^) | *S_r_* (% ºC^-1^) | *δT* (ºC) |
| --- | --- | --- | --- |
| 365 nm LEDs | −0.0210 ± 0.0003 | 2.11 ± 0.03 | 0.06 |
| 385 nm LEDs | −0.0122 ± 0.0002 | 1.22 ± 0.02 | 0.07 |
| White-emitting LEDs | −0.0132 ± 0.0001 | 1.32 ± 0.01 | 0.07 |
| AM1.5G | −0.0029 ± 0.0002 | 0.29 ± 0.02 | 0.3 |

1. **Figures**

**Figure S1**. (a,b) FT-IR spectra and (c,d) XRD patterns of SEBS and SEBS doped with distinct concentrations of Eu- and Tb-based complex, respectively.

**Figure S2**. Room-temperature excitation (left) and emission (right) spectra of Tb-BBA-Phen.

**Figure S3**. Room-temperature excitation (left) and emission (right) spectra of Eu-TFNB-Phen.

**Figure S4.** Room-temperature excitation (left) and emission (right) spectra of SEBS-Eu.

**Figure S5.** Excitation (left) and emission (right) spectra of SEBS-Eu measured at 18 K.

**Figure S6.** Room-temperature excitation (left) and emission (right) spectra of SEBS-Tb.

**Figure S7.** Excitation (left) and emission (right) spectra of SEBS-Tb measured at 18 K.

**Figure S8**. Room-temperature excitation (left) and emission (right) spectra of SEBS.

**Figure S9.** Emission decay curves of Eu-TFNB-Phen excited at 355 nm and monitored at 611 nm measured at 14 K. The solid line represents the data best fit (R^2^ > 0.99), using a single-exponential function $I\left( t \right)=I_{1}e^{-(t-t_{0})/\tau_{1}}$. The emission lifetime $\tau_{1}$ was estimated to be 0.661±0.002 ms. The respective residual plot is shown on the right-hand side.

**Figure S10**. Room temperature emission decay curves of Eu-TFNB-Phen excited at 355 nm and monitored at 625 nm. The solid line represents the data best fit (R^2^ > 0.87), using a single-exponential function $I\left( t \right)=I_{1}e^{-(t-t_{0})/\tau_{1}}$. The emission lifetime $\tau_{1}$ was estimated to be 0.590±0.006 ms. The respective residual plot is shown on the right-hand side.

**Figure S11.** Emission decay curves of Tb-BBA-Phen excited at 355 nm and monitored at 542 nm measured at 14 K. The solid line represents the data best fit (R^2^ > 0.97), using a single-exponential function $I\left( t \right)=I_{1}e^{-(t-t_{0})/\tau_{1}}$. The emission lifetime $\tau_{1}$ was estimated to be 1.175±0.004 ms. The respective residual plot is shown on the right-hand side.

**Figure S12.** Room temperature emission decay curves of SEBS-Eu excited at 355 nm and monitored at 612 nm. The solid line represents the data best fit (R^2^ > 0.99), using a single-exponential function $I\left( t \right)=I_{1}e^{-(t-t_{0})/\tau_{1}}$. The emission lifetime $\tau_{1}$ was estimated to be 0.5442±0.0002 ms. The respective residual plot is shown on the right-hand side.

**Figure S13.** Emission decay curve of SEBS-Eu measured at 18 K excited at 355 nm and monitored at 612 nm. The solid line represents the data best fit (R^2^ > 0.99), using a single-exponential function $I\left( t \right)=I_{1}e^{-(t-t_{0})/\tau_{1}}$. The emission lifetime $\tau_{1}$ was estimated to be 0.639±0.001 ms. The respective residual plot is shown on the right-hand side.

**Figure S14.** Room temperature emission decay curves of SEBS-Tb excited at 355 nm and monitored at 542 nm. The solid line represents the data best fit (R^2^ > 0.99), using a single-exponential function $I\left( t \right)=I_{1}e^{-(t-t_{0})/\tau_{1}}$. The emission lifetime $\tau_{1}$ was estimated to be 0.786±0.001 ms. The respective residual plot is shown on the right-hand side.

**Figure S15.** Emission decay curve of SEBS-Tb measured at 18 K excited at 355 nm and monitored at 542 nm. The solid line represents the data best fit (R^2^ > 0.97), using a single-exponential function $I\left( t \right)=I_{1}e^{-(t-t_{0})/\tau_{1}}$. The emission lifetime $\tau_{1}$ was estimated to be 0.940±0.001 ms. The respective residual plot is shown on the right-hand side.


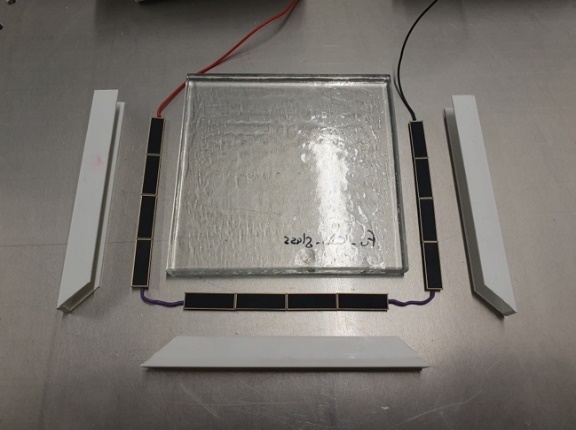

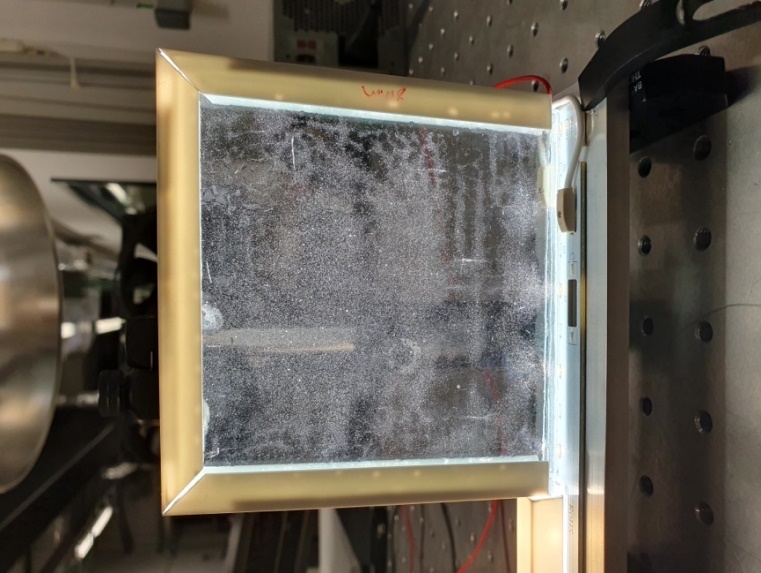


**Figure S16**: Photographs of the (a) prototype assembly with a 12 c-Si solar cell array and PVC frame, and (b) assembled prototype illuminated by 5 white LEDs. The full size of the prototype is 105x105x10 mm^3^.


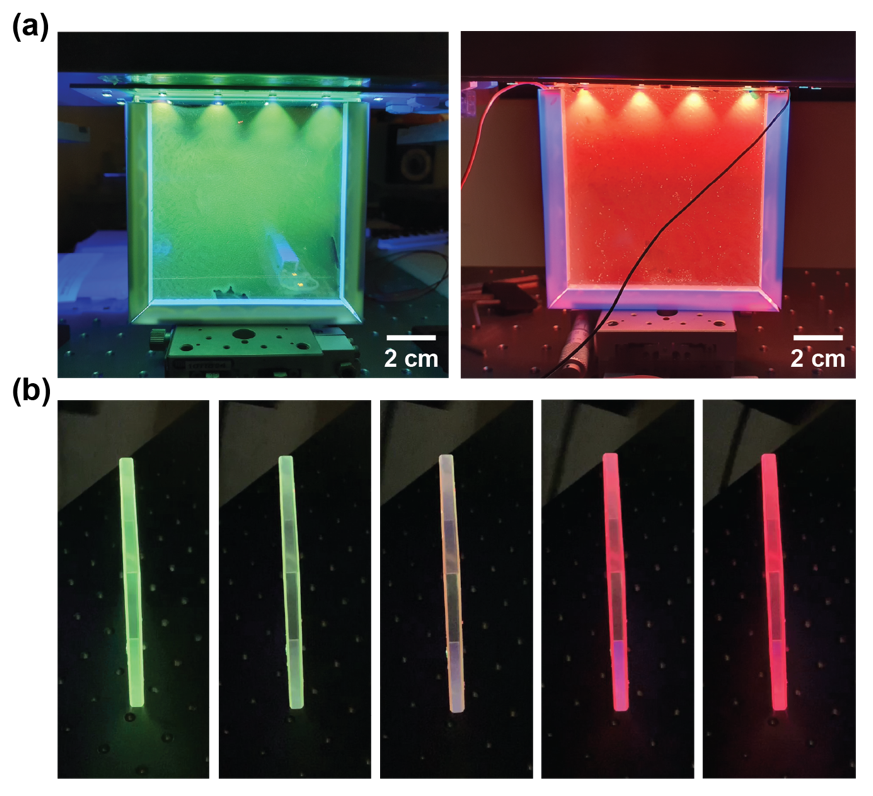


**Figure S17.** (a) Photographs of the LSCs based on SEBS-Tb (left) and SEBS-Eu (right) prototypes under integrated LED irradiation at 340 nm. (b) Sequential frames of video depicting emission of each side of the double-sided LSC under UV radiation at 365 nm (full video available in Supporting Information).

**Figure S18.** Transmittance curve of the LSC based on SEBS-Eu.


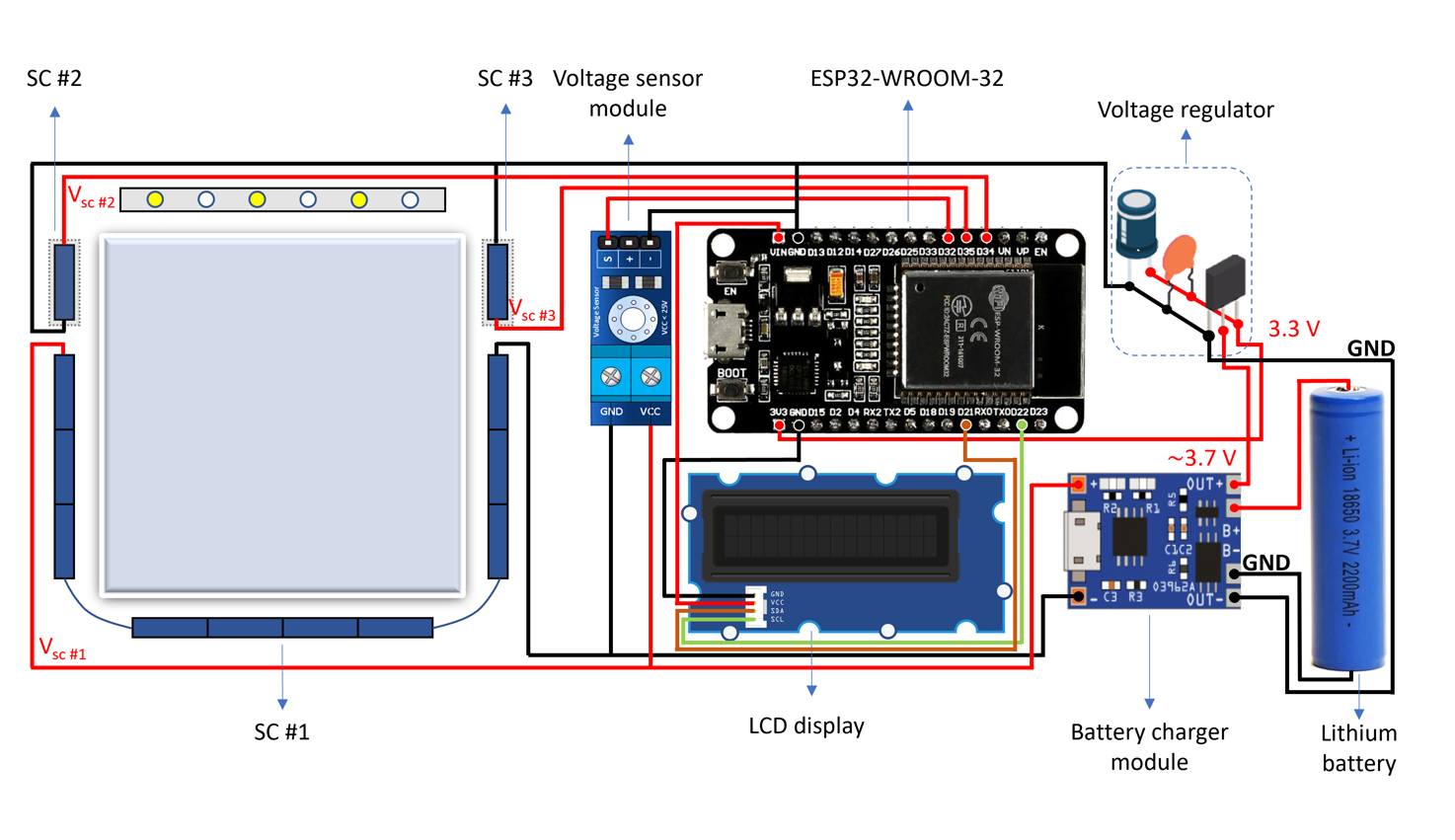


**Figure S19.** Scheme of the IoT platform integrated with the LSC prototype.


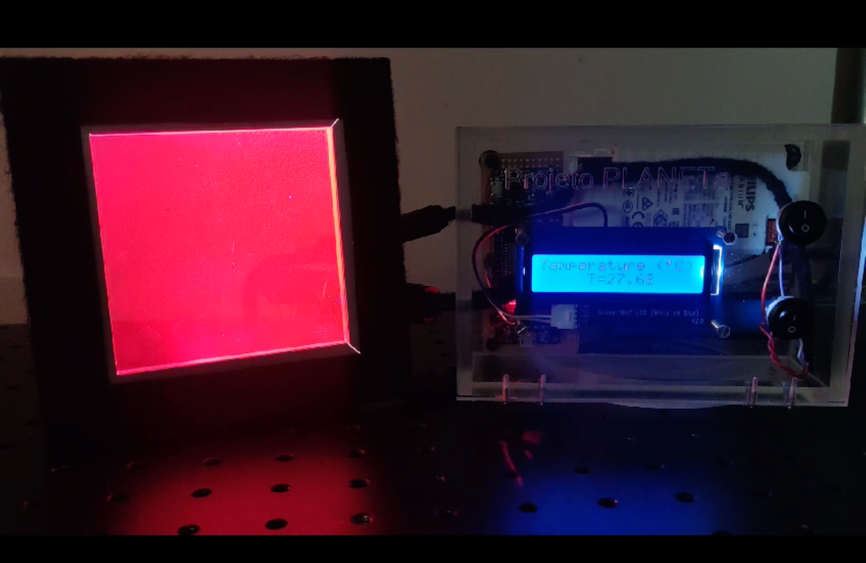


**Figure S20.** Frame of a video showing the prototype based on SEBS-Eu working as temperature sensor using integrated artificial LED lighting (full video available in Supporting Information).


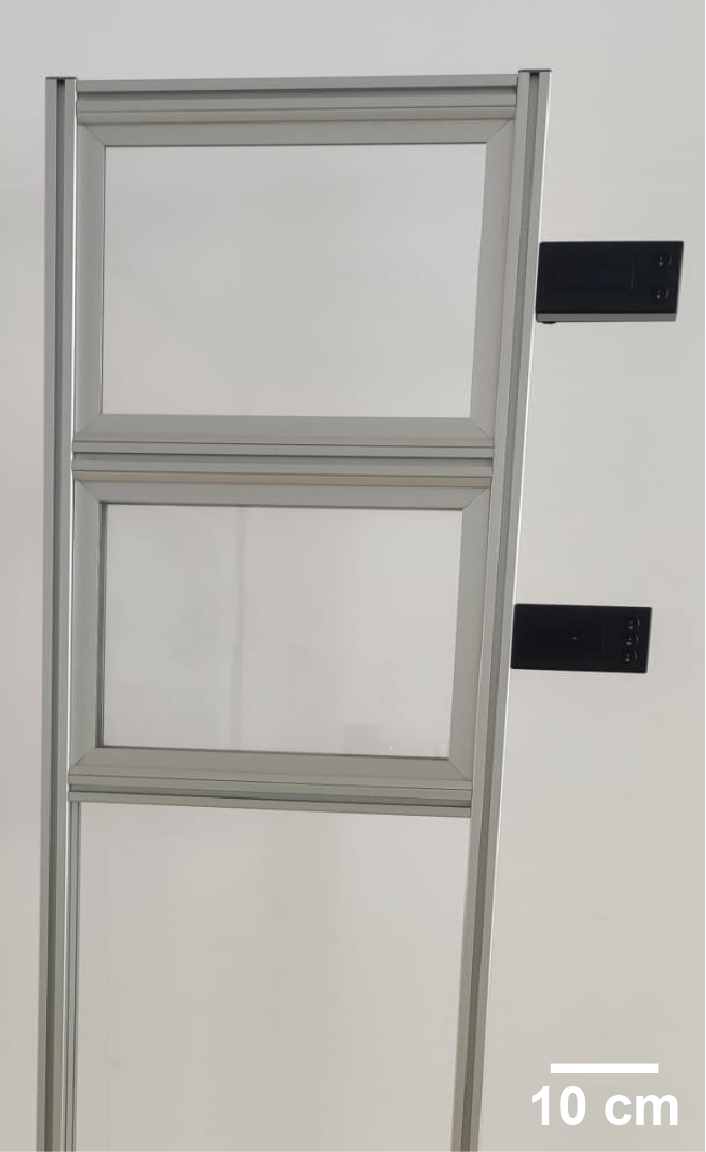


**Figure S21.** Final demonstrator comprising the large-area SEBS-Eu (top) and SEBS-Tb (bottom) prototypes, the artifical LED lighting system and the black boxes where the electronic components are assembled.

**Figure S22**. Emission spectra of the distinct illumination sources used in the prototype’s characterization.


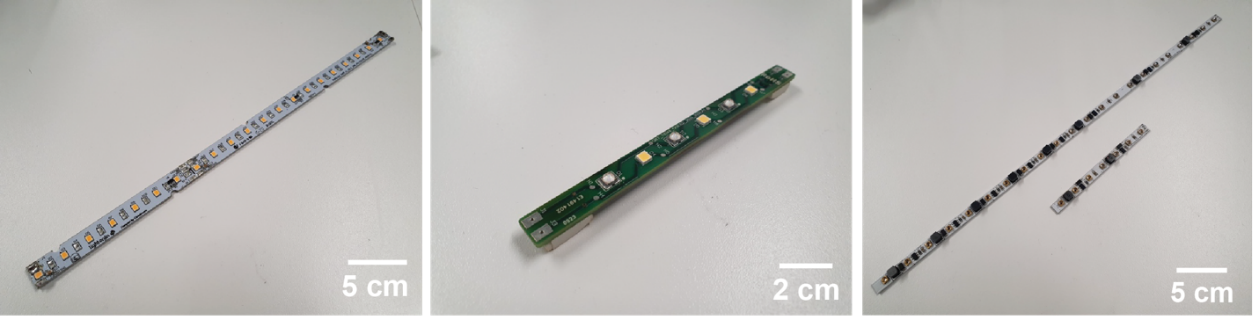


**Figure S23.** LED arrays with white-light emission (left), combination of white and UV radiation at 385 nm (center) and 10 cm and 36 cm array with combination of 255 nm and 365 nm (right).

**References**

[1] J. A. Fernandes, R. A. S. Ferreira, M. Pillinger, L. D. Carlos, J. Jepsen, A. Hazell, P. Ribeiro-Claro, I. S. Goncalves, *J. Lumin.*, **2005**, 113, 50.

[2] R. Reisfeld, D. Shamrakov, C. Jorgensen, *Sol. Energy Mater. Sol. C.*, **1994**, 33, 417.

[3] S. F. H. Correia, P. P. Lima, P. S. André, R. A. S. Ferreira, L. D. Carlos, *Sol. Energy Mater. Sol. C.*, **2015**, 138, 51.

[4] S. A. Wade, S. F. Collins, G. W. Baxter, *J. Appl. Phys.*, **2003**, 94, 4743.

[5] C. D. S. Brites, A. Millán, L. D. Carlos, in *Handbook on the Physics and Chemistry of Rare Earths*, Vol. 49 (Eds: J.-C. Bünzli, V. K. Pecharsky), Elsevier Science, B. V., Amsterdam 2016.

[6] B. L. Zhang, P. J. Zhao, L. J. Wilson, J. Subbiah, H. B. Yang, P. Mulvaney, D. J. Jones, K. P. Ghiggino, W. W. H. Wong, *ACS Energy Lett.*, **2019**, 4, 1839.

[7] F. Mateen, Y. L. Li, M. A. Saeed, Y. J. Sun, Y. C. Zhang, S. Y. Lee, S. K. Hong, *J. Lumin.*, **2021**, 231, 117837.

[8] F. Meinardi, H. McDaniel, F. Carulli, A. Colombo, K. A. Velizhanin, N. S. Makarov, R. Simonutti, V. I. Klimov, S. Brovellii, *Nat. Nanotechnol.*, **2015**, 10, 878.

[9] F. Meinardi, A. Colombo, K. A. Velizhanin, R. Simonutti, M. Lorenzon, L. Beverina, R. Viswanatha, V. I. Klimov, S. Brovelli, *Nat. Photonics*, **2014**, 8, 392.

[10] L. J. Brennan, F. Purcell-Milton, B. McKenna, T. M. Watson, Y. K. Gun'ko, R. C. Evans, *J. Mater. Chem. A*, **2018**, 6, 2671.

[11] F. Meinardi, S. Ehrenberg, L. Dhamo, F. Carulli, M. Mauri, F. Bruni, R. Simonutti, U. Kortshagen, S. Brovelli, *Nat. Photonics*, **2017**, 11, 177.

[12] H. B. Li, K. F. Wu, J. Lim, H. J. Song, V. I. Klimov, *Nat. Energy*, **2016**, 1, 16157.

[13] K. F. Wu, H. B. Li, V. I. Klimov, *Nat. Photonics*, **2018**, 12, 105.

[14] F. Corsini, A. Nitti, E. Tatsi, G. Mattioli, C. Botta, D. Pasini, G. Griffini, *Adv. Opt. Mater.*, **2021**, 9, 2100182.

[15] N. Aste, L. C. Tagliabue, C. Del Pero, D. Testa, R. Fusco, *Renew. Energy*, **2015**, 76, 330.

[16] X. Y. Li, J. Qi, J. Y. Zhu, Y. X. Jia, Y. Liu, Y. R. Li, H. Liu, G. Li, K. F. Wu, *J. Phys. Chem. Lett.*, **2022**, 13, 9177.

[17] C. H. Yang, M. Moemeni, M. Bates, W. Sheng, B. Borhan, R. R. Lunt, *Adv. Opt. Mater.*, **2020**, 8, 1901536.

[18] A. Anand, M. L. Zaffalon, G. Gariano, A. Camellini, M. Gandini, R. Brescia, C. Capitani, F. Bruni, V. Pinchetti, M. Zavelani-Rossi, F. Meinardi, S. A. Crooker, S. Brovelli, *Adv. Funct. Mater.*, **2020**, 30, 1906629.

[19] G. J. Liu, R. Mazzaro, Y. Q. Wang, H. G. Zhao, A. Vomiero, *Nano Energy*, **2019**, 60, 119.

[20] H. G. Zhao, *J. Lumin.*, **2019**, 211, 150.

[21] G. J. Liu, H. G. Zhao, F. Y. Diao, Z. B. Ling, Y. Q. Wang, *J. Mater. Chem. C*, **2018**, 6, 10059.

[22] H. G. Zhao, D. Benetti, X. Tong, H. Zhang, Y. F. Zhou, G. J. Liu, D. L. Ma, S. H. Sun, Z. M. M. Wang, Y. Q. Wang, F. Rosei, *Nano Energy*, **2018**, 50, 756.

[23] H. G. Zhao, R. J. Sun, Z. F. Wang, K. F. Fu, X. Hu, Y. H. Zhang, *Adv. Funct. Mater.*, **2019**, 29, 1902262.

[24] Y. L. Li, J. Olsen, K. Nunez-Ortega, W. J. Dong, *Sol. Energy*, **2016**, 136, 668.

[25] S. J. Gallagher, B. Norton, P. C. Eames, *Sol. Energy*, **2007**, 81, 813.

[26] H. G. Zhao, G. J. Liu, G. T. Han, *Nanoscale Adv.*, **2019**, 1, 4888.

[27] H. G. Zhao, G. J. Liu, S. J. You, F. V. A. Camargo, M. Zavelani-Rossi, X. H. Wang, C. C. Sun, B. Liu, Y. M. Zhang, G. T. Han, A. Vomiero, X. Gong, *Energy Environ. Sci.*, **2021**, 14, 396.

[28] B. X. Liu, L. H. Wang, X. Gong, H. G. Zhao, Y. M. Zhang, *J. Mater. Chem. C*, **2022**, 10, 18154.

[29] J. R. Li, H. G. Zhao, X. J. Zhao, X. Gong, *Nanoscale Horiz.*, **2023**, 8, 83.

[30] X. Gong, S. Y. Zheng, X. J. Zhao, A. Vomiero, *Nano Energy*, **2022**, 101, 107617.

[31] Y. Han, X. J. Zhao, A. Vomiero, X. Gong, H. G. Zhao, *J. Mater. Chem. C*, **2021**, 9, 12255.

[32] J. R. Li, H. G. Zhao, X. J. Zhao, X. Gong, *Nanoscale*, **2021**, 13, 9561.

[33] Y. M. You, X. Tong, A. I. Channa, H. Q. Zhi, M. K. Cai, H. Y. Zhao, L. Xia, G. J. Liu, H. G. Zhao, Z. M. Wang, *Chem. Eng. J.*, **2023**, 452, 139490.

[34] T. A. de Bruin, R. Terricabres-Polo, A. Kaul, N. K. Zawacka, P. T. Prins, T. F. J. Gietema, A. C. de Waal, D. K. G. de Boer, D. A. M. Vanmaekelbergh, P. Leblans, S. Verkuilen, Z. Hens, C. D. Donega, W. G. J. H. M. van Sark, *Sol. RRL*, **2023**, 7, 2201121.

[35] G. J. Liu, M. Zavelani-Rossi, G. T. Han, H. G. Zhao, A. Vomiero, *J. Mater. Chem. A*, **2023**, 11, 8950.

[36] L. R. Wilson, E. Klampaftis, B. S. Richards, *IEEE J. Photovolt.*, **2017**, 7, 802.

[37] W. van Sark, P. Moraitis, C. Aalberts, M. Drent, T. Grasso, Y. L'Ortije, M. Visschers, M. Westra, R. Plas, W. Planje, *Sol. RRL*, **2017**, 1, 1600015.

[38] F. Mateen, N. Lee, S. Y. Lee, S. T. U. Din, W. Yang, A. Shahzad, A. K. Kaliamurthy, J. J. Lee, S. K. Hong, *Polymers*, **2021**, 13, 3770.

[39] P. Meti, F. Mateen, D. Y. Hwang, Y. E. Lee, S. K. Hong, Y. D. Gong, *Dyes Pigm.*, **2022**, 202, 110221.

[40] K. B. Cai, H. Y. Huang, M. L. Hsieh, P. W. Chen, S. E. Chiang, S. H. Chang, J. L. Shen, W. R. Liu, C. T. Yuan, *ACS Nano*, **2022**, 16, 3994.

[41] J. Huang, J. J. Zhou, E. Jungstedt, A. Samanta, J. Linnros, L. A. Berglund, I. Sychugov, *ACS Photonics*, **2022**, 9, 2499.

[42] S. S. Han, G. Chen, C. H. Shou, H. Peng, S. L. Jin, C. C. Tu, *ACS Appl. Mater. Interfaces*, **2020**, 12, 43771.

[43] S. Li, H. C. Liu, W. Chen, Z. M. Zhou, D. Wu, R. Lu, B. X. Zhao, J. J. Hao, L. Yang, H. C. Yang, R. Cai, B. Xu, K. Wang, X. W. Sun, *Sol. Energy Mater. Sol. C.*, **2020**, 206, 110321.

[44] C. K. Gordon, R. F. Hogg, M. W. Brett, L. D. Browne, D. M. de Clercq, M. B. Price, N. J. L. K. Davis, *J. Phys. Chem. C*, **2022**, 126, 19803.

[45] L. Dhamo, F. Carulli, P. Nickl, K. D. Wegner, V. D. Hodoroaba, C. Wurth, S. Brovelli, U. Resch-Genger, *Adv. Opt. Mater.*, **2021**, 9, 2100587.

[46] D. C. J. Neo, W. P. Goh, H. H. Lau, J. Shanmugam, Y. F. Chen, *ACS Appl. Nano Mater.*, **2020**, 3, 6489.

[47] N. S. Makarov, D. Korus, D. Freppon, K. Ramasamy, D. W. Houck, A. Velarde, A. Parameswar, M. R. Bergren, H. McDaniel, *ACS Appl. Mater. Interfaces*, **2022**, 14, 29679.

[48] M. Y. Wei, F. P. G. de Arguer, G. Walters, Z. Y. Yang, L. N. Quan, Y. Kim, R. Sabatini, R. Quintero-Bermudez, L. Gao, J. Z. Fan, F. J. Fan, A. Gold-Parker, M. F. Toney, E. H. Sargent, *Nat. Energy*, **2019**, 4, 197.

[49] Z. L. Li, A. Johnston, M. Y. Wei, M. I. Saidaminov, J. M. de Pina, X. P. Zheng, J. K. Liu, Y. Liu, O. M. Bakr, E. H. Sargent, *Joule*, **2020**, 4, 631.

[50] J. J. Wu, J. Y. Tong, Y. Gao, A. F. Wang, T. Zhang, H. R. Tan, S. M. Nie, Z. T. Deng, *Angew. Chem. Int. Edit.*, **2020**, 59, 7738.

[51] L. Zdrazil, S. Kalytchuk, M. Langer, R. Ahmad, J. Pospisil, O. Zmeskal, M. Altomare, A. Osvet, R. Zboril, P. Schmuki, C. J. Brabec, M. Otyepka, S. Kment, *ACS Appl. Energy Mater.*, **2021**, 4, 6445.
